# Draft Genome Sequence of Methicillin-Resistant Staphylococcus aureus Harboring Staphylococcal Cassette Chromosome *mec* Type IX, Isolated from a Fatal Bacteremic Pneumonia Case

**DOI:** 10.1128/MRA.00616-21

**Published:** 2021-07-29

**Authors:** Peechanika Chopjitt, Thidathip Wongsurawat, Piroon Jenjaroenpun, Parichart Boueroy, Watcharaporn Kamjumphol, Rujirat Hatrongjit, Anusak Kerdsin

**Affiliations:** a Faculty of Public Health, Kasetsart University, Chalermphrakiat Sakon Nakhon Province Campus, Sakon Nakhon, Thailand; b Division of Bioinformatics and Data Management for Research, Department of Research and Development, Faculty of Medicine Siriraj Hospital, Mahidol University, Bangkok, Thailand; c National Institute of Health, Department of Medical Sciences, Ministry of Public Health, Nonthaburi, Thailand; d Faculty of Science and Engineering, Kasetsart University, Chalermphrakiat Sakon Nakhon Province Campus, Sakon Nakhon, Thailand; University of Maryland School of Medicine

## Abstract

Here, we report the whole-genome sequence of a methicillin-resistant Staphylococcus aureus strain harboring staphylococcal cassette chromosome *mec* (SCC*mec*) type IX, isolated from a fatal bacteremic pneumonia case. Genomic analysis revealed that the isolate was sequence type 9 and *spa* type t3446, carrying multiple antimicrobial resistance genes comprising *mecA*, *blaZ*, *aac(6′)-aph(2″)*, *aadD*, *ant(6)-Ia*, *lsa*(E), *dfrG*, *tet*(M), *fexA*, and *lnu*(B).

## ANNOUNCEMENT

Methicillin-resistant Staphylococcus aureus (MRSA) strains are classified as hospital-acquired (HA), community-acquired (CA), and livestock-associated (LA) infections (1). MRSA strains carry different types of the staphylococcal cassette chromosome *mec* (SCC*mec* I to XIII). While SCC*mec* I to III are commonly found in HA-MRSA, SCC*mec* IV to XIII are usually detected in CA-MRSA and LA-MRSA (2).

Interestingly, MRSA sequence type 9 (ST9) harboring SCC*mec* IX was reported in humans as a newly identified CA-MRSA clone disseminating in Thailand (3). Herein, we determined the genome sequence of the MRSA strain carrying SCC*mec* IX (isolate M16), isolated in February 2019 from sputum from a 49-year-old man in northern Thailand with a fatal case of bacteremic pneumonia. The isolate was cultured on sheep blood agar at 37°C for 18 h and identified using conventional biochemical tests (4). Its resistance to methicillin was investigated using cefoxitin disk diffusion according to the 2020 Clinical and Laboratory Standard Institute (CLSI) guidelines (5). A pentaplex PCR assay was used to simultaneously identify the genus (Staphylococcus; 16S rRNA), the species (S. aureus; *femA*), and the methicillin resistance (*mecA*) and PVL toxin (*lukS*) genes (6). These assays demonstrated that the isolate was a MRSA strain with no *lukS* gene.

The bacterium was grown on tryptic soy agar at 37°C for 18 h. Genomic DNA was extracted from the colony using a ZymoBIOMICS DNA kit (Zymo Research, USA) and quantified using the Invitrogen Qubit double-stranded DNA (dsDNA) high-sensitivity (HS) assay kit (Thermo Fisher Scientific, MA, USA). Genomic libraries were generated using the NEBNext Ultra II DNA library prep kit for Illumina (New England BioLabs, USA) following the manufacturer’s instructions. Whole-genome sequencing was performed using the MiSeq platform (Illumina, CA, USA) according to the manufacturer’s instructions to obtain 250-bp paired-end reads (7). We applied Skewer v0.2.2 (8) for quality filtering and adapter trimming of the Illumina reads. Quality checking of the Illumina reads was performed using FastQC v0.11.8 (https://www.bioinformatics.babraham.ac.uk/projects/fastqc/), and the genome was *de novo* assembled using Unicycler v0.4.8 (9). The genome sequences were checked for quality using QUAST v5.0.2 (10). The genomic sequences were submitted to the NCBI Prokaryotic Genome Annotation Pipeline (PGAP) v4.12 for annotation. Default parameters were used for all software unless otherwise specified.

In total, 4,242,022 raw reads were obtained for isolate M16. Sixty-nine contigs were assembled, with an *N*_50_ value of 126,866 bp. On average, the assembled draft genome sequence was covered 230.42 times. The draft genome size was determined to be 2,761,167 bp and the GC content to be 32.72%. This isolate was identified as ST9, carried SCC*mec* IX, and had the *spa* type t3446 according to MLST 2.0, SCC*mec*Finder, and spaTyper 1.0, respectively (11–13). The arginine catabolic mobile element (ACME) was not detected using MyDbFinder (https://cge.cbs.dtu.dk/services/MyDbFinder/). The isolate genome included acquired antimicrobial resistance genes, namely, *mecA*, *blaZ*, *aac(6′)-aph(2″)*, *aadD*, *ant(6)-Ia*, *lsa*(E), *dfrG*, *tet*(M), *fexA*, and *lnu*(B), according to ResFinder v4.1 (14). We detected *tet*(M) located on the *mecA* contig, which suggested that it was carried on the *SCCmec* IX element. The VirulenceFinder tool revealed aureolysin (*aur*), enterotoxin types G, I, M, N, O, and U (*seg*, *sei*, *sem*, *sen*, *seo*, and *seu*), and γ-hemolysin (*hlgA*, *hlgB*, and *hlgC*) (15).

This study was reviewed and approved by the Ethics Review Board (ERB) of the Ministry of Public Health, Thailand. The ERB waived the requirement for informed consent because the study satisfied the conditions of the policy statement on ethical conduct for research involving humans. This study was conducted according to the principles of the Declaration of Helsinki.

### Data availability.

The results of this whole-genome shotgun project were deposited in DDBJ/ENA/GenBank under the BioProject accession no. PRJNA735605, BioSample accession no. SAMN19589956, and accession no. JAHKSK000000000.1. The Sequence Read Archive (SRA) number is SRR14802804.
